# Holding back the genes: limitations of research into canine behavioural genetics

**DOI:** 10.1186/2052-6687-1-7

**Published:** 2014-06-10

**Authors:** Diane van Rooy, Elizabeth R Arnott, Jonathan B Early, Paul McGreevy, Claire M Wade

**Affiliations:** Faculty of Veterinary Science, University of Sydney, Sydney, NSW 2006 Australia

**Keywords:** Behaviour, Dog, Genetics, Heritability

## Abstract

Canine behaviours that are both desirable and undesirable to owners have a demonstrable genetic component. Some behaviours are breed-specific, such as the livestock guarding by maremmas and flank sucking seen in Dobermanns. While the identification of genes responsible for common canine diseases is rapidly advancing, those genes underlying behaviours remain elusive. The challenges of accurately defining and measuring behavioural phenotypes remain an obstacle, and the use of variable phenotyping methods has prevented meta-analysis of behavioural studies. International standardised testing protocols and terminology in canine behavioural evaluations should facilitate selection against behavioural disorders in the modern dog and optimise breeding success and performance in working dogs. This review examines the common hurdles faced by researchers of behavioural genetics and the current state of knowledge.

## Lay summary

All canine behaviour, whether desirable or undesirable to owners, has a genetic component. Studies of “showing eye” and “bark” which compared the behaviour of offspring with parents concluded that behaviour does not follow simple Mendelian inheritance. Some behaviours are breed-specific, such as “livestock guarding” by maremmas and “flank sucking” seen in Dobermanns.

Identification of genes responsible for canine monogenic diseases (caused by one gene) is advancing rapidly, but the genes underlying behaviours remain elusive. This is because canine behaviours are similar to complex diseases such as Hypothyroidism, where there are both environmental and multiple genetic components.

A major obstacle in behavioural studies has been the challenge of accurately defining and measuring behaviours, and how these are expressed (behavioural phenotypes). As researchers have used a variety of different ways to measure behaviour, data from multiple behavioural studies cannot be combined. International standardised testing protocols and terminology (definitions) in canine behavioural evaluations should help selection against behavioural disorders in the dog, and optimise breeding success and performance in working dogs.

This review examines the common hurdles faced by researchers of behavioural genetics and the current state of knowledge.

## Introduction

In the space of 30,000 years, dogs have moved from camp-side scavengers to being considered ‘man’s best friend’. Current statistics on pet ownership show that 36% of both Australian [1] and U.S. households [2] and 23% of UK households [3] own at least one dog. This equates to more than 81 million dogs in those three countries alone.

Since their domestication, dogs have played many roles in their association with humans. For hundreds of years they have been both companions and valuable working colleagues, assisting us with protection, transport, agricultural production and hunting. While domestic dogs retain many of the behavioural vestiges of their wolf ancestors, both their morphology and behaviour have been greatly altered by artificial selection. These changes were often distilled by geographic isolation. In the 19^th^ century, extensive record keeping began to trace the lineages of dogs bred for purpose. The domesticated dog became refined into breeds with closed studbooks and written breed standards. As leisure time and wealth increased, the objectives of selection altered from being purpose-driven to fashion-driven. Although some working breeds are still in demand, and some utilise their original working traits in contemporary canine sporting activities such as agility trials, numerous breeds have been selected for purposes unrelated to practical work. In the role of companions, modern dogs may require higher tolerance than their ancestors for the frustration of inactivity, social isolation and unstimulating environments [4]. In this sense, the companion dog may be regarded as an evolutionary work in progress [5], proving highly adaptable and malleable to human needs, but the niche into which dogs must evolve continues to shift.

The formation of dog breeds has created a genome that is extremely well suited to genetic research. There is large genetic variation between breeds and small variation within breeds [6]. Linkage disequilibrium may extend for megabases within some breeds, compared with only tens of kilobases in humans, making genome-wide studies more economical in dogs [7]. With advances in genomic biology [6, 8, 9], it is not surprising that researchers have been keen to map the genes that allow dog breeds to express the specific behaviours for which they are so well known [10, 11]. Frustratingly, this has been less successful than initially hoped, and greater gene mapping success may result from deconstructing factor-based phenotypes into their underlying components [12].

The sequencing of the canine genome in 2005 heralded an escalation in the development of genetic tests. The Canine Health Foundation website currently lists 102 genetic tests. Of these, 97 are tests for inherited diseases and five are concerned with coat characteristics (http://www.caninehealthfoundation.org). It is worth asking why so much progress has been made in canine inherited diseases and so little in the genetics of canine behaviours. Improved understanding of canine behavioural genetics has the potential to benefit the dogs themselves and provide useful models for several human psychiatric disorders.

This paper will focus on the limitations and hurdles faced by researchers in the field of canine behavioural genetics. It also includes a discussion of behavioural traits for which a genetic basis is already understood, or currently being studied.

## Review

### Challenges of canine behavioural genetics

#### Complexity of behaviour

An animal’s behaviour is influenced by inheritance and interactions between behaviours, the environment, learning and epigenetics. Consequently, these interactions must be considered when attempting to identify the genetic contribution.

##### Polygenic inheritance

Even with the vastly improved genetic tools now available to researchers, gene mapping of complex traits is considerably more difficult than mapping Mendelian traits. The International Schizophrenia Consortium [13] proposed that, rather than a few genes having a large effect, schizophrenia and bipolar disorders in humans are most likely influenced by a large number of loci that are collectively responsible for variation in risk.

Similarly, a complex mode of inheritance has long been suspected for behavioural traits of interest in working dogs. Kelley [14] and Scott [15] investigated the inheritance of the herding and hunting traits ‘showing eye’ and ‘bark’ , respectively. By comparing the behaviour of progeny to parents, both authors concluded these behaviours do not follow a simple Mendelian pattern of inheritance.

##### Interactions with environment and learning

While this paper focuses on the influence of genes on behaviour, the enormous impact of environment, both the current environment and the lifetime experiences of the dog, cannot be overlooked [16]. Genes code for proteins, not disorders. The behaviour results from the complex ongoing interactions between these proteins and the environment. All behaviours must be viewed in the environmental context in which they are occurring. Learning plays a vital role – dogs will repeat behaviours that were successful in the past. It is thought genetics may influence a dog’s predisposition to a behavioural disorder in a number of ways: how information about potential threats is detected and interpreted, how memories of past experiences are used, or by altering the metabolism of neurotransmitters.

Early experiences in the lives of dogs can affect their development and future behaviour. For example, dogs who experienced an illness in the early part of their life were significantly more likely to exhibit owner-defined behavioural problems later in life [17]. These behavioural problems included aggression and fear towards strangers, separation-related barking and inappropriate sexual behaviour. Deprivation of essential nutrients in early life may also have long-lasting effects. For example, a diet deficient in the polyunsaturated fatty acids that are necessary for early brain development may affect associative learning and cognition [18]. Finally, behaviours of offspring may be influenced by epigenetic mechanisms. Maternal behaviours alter the methylation of DNA in the offspring, thereby affecting gene expression in future generations [19].

##### Interactions between behaviours

Dogs are frequently afflicted by more than one anxiety disorder, suggesting a common biological basis. For instance, dogs diagnosed with noise or thunderstorm phobias have a high probability (0.88 and 0.86, respectively) of also demonstrating separation anxiety [20]. There is also co-morbidity among different categories of aggression, while anxiety disorders and aggression disorders have likewise been shown to be linked [21]. These behavioural interactions add to the complexity of defining a behavioural phenotype.

#### Phenotyping

Phenotyping must be valid, reliable, sensitive and as objective as possible to be useful for genetic analysis. Unlike most diseases studied, there are no specific physical characteristics or blood tests for behavioural conditions. Clinical criteria used for phenotyping may overlap or be either more restrictive or broader than the criteria used for diagnosis. Methods of phenotyping used in behavioural studies include battery testing, owner questionnaires and observational study. The latter is less common due to financial and time costs.

##### Testing

Numerous behavioural tests are applied to dogs. Some measure only a single trait: for example, the Ainsworth’s Strange Situation Test measures the attachment between dog and owner. Others measure different aspects of temperament or aptitude for a particular function. In their review of the behavioural testing methodology used in over 30 studies, Diederich and Giffroy [22] found a widespread lack of standardisation for most parameters: age of testing; site of testing (indoor/outdoor); social stimuli used (caged dogs, free-range dogs); and, especially, environmental stimuli used. For example, auditory stimuli (strong, prolonged noise) varied from a clock alarm, siren, doorbell, or whistle to a vacuum cleaner. In a similar vein, a lack of standardisation in canine laterality tests has also been reported [23]. Although numerous behavioural studies are being undertaken, the use of different methodologies limits meta-analysis and potential progress.

The Dog Mentality Assessment, one of the more common standardised behavioural tests available, is used to test thousands of Swedish dogs each year. Comparing test results with owner questionnaire responses, it appears to reliably measure playfulness, sociability and curiosity/fearlessness and the boldness-shyness personality dimension [24]. However, results can also be affected by external factors. While the boldness-shyness dimension accounted for over half the additive variation in a cohort of more than 10,000 German Shepherds and Rottweilers [25], the effect of the judge scoring the dogs was highly significant. This supports the findings of Ruefenacht [26] that heritability of traits such as self-confidence, defence drive and hardness in German Shepherds was significantly affected by the sex and age of the dog, the kennel the dog came from and the judge used for scoring these traits.

Livestock breeding programmes demonstrate that using objective trait testing procedures is critical in establishing successful breeding programmes for complex traits [27]. The importance of objectivity was highlighted in a recent study of the heritability of herding behaviour in dogs [28]. Herding phenotypes assessed in the study included ‘eye’ , ‘balance’, ‘bark’ and ‘power’. The authors used the results of the Swedish Sheepdog Society’s standardised Herding Trait Characterisation tests from 1989 to 2003. The test was revised in 1996 to be a more subjective assessment. Heritabilities of the majority of traits were higher when the original, more objective neutral descriptors were used. For example, the original ‘effective working distance’ trait initially had an estimated heritability of 0.50, whereas the revised version had an estimated heritability of 0.18. This shift emphasises the sensitivity of heritability measures to confounding factors [28, 29].

Statistical analysis of performance recording data needs to correct phenotypic information for known environmental factors. In two studies of Finnish hunting dogs, weather conditions, such as the presence of wind and snow, and the month the trial was held significantly affected performance [30, 31]. Liinamo et al. [31] attributed the low repeatabilities and estimated heritabilities of most hunting traits to the large effect that environmental variation had on the results. The authors proposed that best-linear unbiased predictor-based estimated breeding values would be a preferred means of taking these environmental differences into account.

##### Questionnaires

Owner-based questionnaires have several advantages over battery testing. The assessor is intimately familiar with the subject and can assess behaviour over numerous events compared to assessment based on a single trial. The assessment is also carried out in the home environment of the dog, rather than the artificial environment of a testing area. Because questionnaires are relatively economical, they are commonly used in research.

The Canine Behavioural Assessment and Research Questionnaire (C-BARQ) is a validated questionnaire that has been used in several studies [32–35]. Owners assess either the frequency or severity of numerous behaviours in a variety of situations using a 5-point ordinal scale. Questions are then scored, grouped and averaged to give scores for 14 behavioural factors. Similarly, for the validated Monash Canine Personality Questionnaire-Revised (MCPQ-R), owners give a rating between one and six for 26 adjectives of personality traits. The MCPQ-R measures five personality dimensions of dogs: extraversion, motivation, training focus, amicability and neuroticism. Test-retest and inter-rater reliabilities for both C-BARQ and MCPQ-R are acceptable [36, 37]. When phenotyping aggression in golden retrievers, the authors proposed C-BARQ as a useful phenotyping technique, second only to personal interview with the owner, and more reliable than battery testing [35].

The main disadvantages of owner-based questionnaires are possible reductions in validity, reliability and objectivity. Each question must be validated to ensure it is measuring the behaviour in question. The large number of assessors will affect inter-assessor reliability. As each participant has a different assessor, there is the risk of each placing a slightly different interpretation on the question and on the dog’s behaviour. One questionnaire designed to reduce this subjectivity is being used to phenotype dogs for a genome-wide association analysis of noise phobias and anxieties [38]. Owners are asked to select which response(s), from a list of possibilities, their dog shows in specific circumstances. Information about frequency, severity and intensity of the response is then combined.

Again, meta-analysis of canine behavioural research projects is difficult as so few studies use the same questionnaire for phenotyping. More progress could be made if behavioural studies could be directly compared and results pooled. Developing a manual of standardised canine phenotyping techniques, containing both testing procedures and questionnaires, would greatly assist progress and be a very useful resource for researchers.

#### Terminology

Inconsistency in terminology is recognised as a major handicap to advancing behavioural science and was the subject of a round-table discussion among 15 international veterinary behaviourists [39]. While participants agreed on the difference between behavioural descriptions and behavioural diagnoses, there was divergence in their approach to including the ‘emotional state’ of the animal and the role of ‘motivation’. Lack of consensus was also identified when a group of behaviourists in the USA was asked to label the scenario of a dog growling when people approached its food bowl. The group members used a variety of terms such as ‘resource guarding’, ‘possessive aggression’ and ‘food-related aggression’ [40].

In its simplest form, an aggressive response tells us that the dog has been pushed to defend its resources, its pups or itself [41]. Nevertheless, aggression is traditionally classified either by the target of the aggression (e.g., owner-directed aggression or dog-directed aggression), or by ascribing the dog’s motivation for the aggression (e.g., redirected aggression or territorial aggression). Each sub-type of aggression, with the exception of pain-related aggression, may well have a distinct genetic basis [42, 43]. While having clear and consistent diagnostic criteria is essential to ensure that we are all talking about the same condition, it may limit investigation into aetiology if conditions are grouped together based only on clinical signs.

Fears, anxiety disorders and phobias are often grouped together and can show similar physiological signs typical of heightened arousal. Both fearful and anxious responses are essential for survival, allowing the dog to avoid the threat currently and in the future [44, 45]. That said, fear and anxiety do differ: fear triggers an immediate response to a perceived threat while anxiety is the anticipation of a perceived threat. The neural pathways also differ: the fearful response is initiated in the amygdala which triggers the sympathetic nervous response and then involves the cerebral cortex (‘bottom up’) while anxiety is controlled by the cerebral cortex which processes the potential threat and later involves the hippocampus (‘top down’) [44]. By grouping fear and anxiety together, we risk incorrectly assuming that there are similar underlying mechanisms and genetic pathways.

##### Behaviour problems or behavioural disorders

Another major difficulty for behavioural researchers is that the term ‘behaviour problems’ has been used to encompass behavioural disorders as well as normal dog behaviours that the owner sees as a problem. Dogs are considered to have a behavioural disorder when their behavioural responses interfere with the dog’s normal function, are persistent, out of proportion to the stimuli, or are triggered by harmless stimuli or a non-existent threat. Behavioural disorders can be due to both maladaptive and pathological/malfunctional causes [46].

Regardless of cause, the bond between dog and owner will become strained if there is a significant divergence between the dog’s behaviour and the expectations of the owner. Behavioural problems are a common reason for relinquishment and it is estimated that they account for 10-15% of all euthanasias of dogs and cats in North America [47] and 21% in Denmark [48]. More recently, O’Neill et al., [49] have reported that behaviour problems are cited in electronic patient records as the most common cause of death in young dogs. Salmon et al. [50] found that, from almost 2,000 dogs surrendered to shelters in the USA, 26% were surrendered solely due to behaviour problems and 40% had a behavioural problem listed as a reason for relinquishment. However, no distinction was made between problem behaviours that are adaptive and may respond well to treatment, and malfunctional pathology. Unfortunately, the associations between pathophysiology and problem behaviour have been reported only in a very limited number of scientific reports, or are largely theoretical, based on analogues from the human literature [51, 52]. In the Danish study [48], 21.4% of 2,493 dogs were euthanased for behavioural reasons and 56.5% of these were due to aggression. Treatment was attempted in only 16% of the dogs and fewer than 5% were referred to a veterinary behaviour practice; the majority of those dogs may not have even received a diagnosis. Aggression was the presenting complaint in 70% of the 1,644 dogs referred to the Animal Behaviour Clinic at Cornell University over 10 years [21]. Anxiety disorders and phobias were the second most common presenting complaint.

#### International collaboration

International sharing of information and pooling of data would greatly increase the power of association studies that are currently limited by small numbers of cases and controls. This is demonstrated by the impressive progress of the Autism Sequencing Consortium since its formation in 2010. Whole-exome sequencing of 1,000 families has identified six genes associated with Autism Spectrum Disorder: *chd8*, *dyrk1a*, *grin2b*, *katnal2*, *pogz* and *scn2a*. With access to data on up to 10,000 families, the consortium aims to use a combination of whole-genome sequencing and whole-exome sequencing to identify and validate further at-risk genes and clarify genotype-phenotype relationships [53]. Data sharing and collaborative research programmes already occur when studying production animals such as cattle [54], pigs [55] and poultry [56].

In 2012, the Swedish Kennel Club hosted a meeting of stakeholders to improve international efforts in the management of canine inherited disorders (Dog Health Workshop, Stockholm, Sweden June 2–3, 2012). Fearful behaviour was identified as the most globally detrimental behaviour across the breeds. Attendees developed plans for both national and international genetic evaluation programmes for dogs [57, 58]. Such programmes could include standardised temperament evaluation tests in addition to screening for important canine health traits, such as hip and elbow dysplasia. This would almost certainly be of enormous value in enabling breeders to produce companion and working dogs most suited to their purpose and potentially lead to a reduction in fear-based behaviours.

### Current knowledge

#### Breed-specific behaviours

Many behaviours seen in domestic dog breeds do exist in their wolf ancestors, but artificial selection has refined and exaggerated some desirable companion qualities and specialist working skills required by owners. For example, pointing (raising a paw in attending to prey) is part of the wolf hunting ethogram that we have intensified in gundogs to alert handlers to the presence of game. As a result of selection, several dog breeds have been developed to show other elements of the lupine hunting ethogram [59]. Herding breeds, such as border collies and Australian cattle dogs, express aspects of hunting behaviour, such as stalking and chasing, to control the movement of livestock, while inhibiting the consummative stages of the hunting sequence. In contrast, maremmas, kuvasz and other guarding breeds live among livestock without showing any hunting or play behaviour.

Breed predilections for behaviour were first described in a long-term project looking at the genetics of social behaviour of dogs [16]. Five breeds were examined over several years in multi-generational pedigrees: basenji, beagle, wire-haired fox terrier, American cocker spaniel and Shetland sheepdog. Breeds differed significantly in their fear of humans – a trait labelled ‘tameness/wildness’. Handling tests produced fear in 100% of basenji pups but in only 38% of cocker spaniel pups. This landmark study also showed statistically significant differences between the levels of playful aggression and so-called dominance traits between the different breeds: wire-haired fox terriers were the most aggressive breed and cocker spaniels the least.

This overt variation in behaviour between breeds has been observed in many subsequent studies. When more than 13,000 dogs belonging to 31 breeds were subjected to the Swedish Dog Mentality Assessment, the aggression scores varied significantly between breeds, although there was also high variance within breeds [60]. Aggression subscores also showed significant differences between 33 breeds when C-BARQ was utilised for phenotyping [33]. A higher proportion of dachshunds, Chihuahuas and Jack Russell terriers showed serious aggression to humans, whereas serious aggression towards unfamiliar dogs was reported in more than 20% of the Akitas, Jack Russell terriers and those dogs classified as pit bull terriers.

More recently, genetic researchers have favoured molecular methods to look for the genes underlying behavioural disorders. The aim of the molecular approach is to identify a potential genetic test that either helps breeders avoid the expression of the phenotype in their pups, or informs better treatment options. Success to date has been hampered by using genetic marker arrays designed for within-breed genetic mapping [8] in an across-breed mapping context. Despite this, indicative association signals have been described for pointing and herding [10] and genotyping by sequencing may further elucidate these traits. We expect that, in traits controlled by many genes, selection and drift will tend to lead to the random fixation of these genes. Gene mapping such complex behaviours and disorders relies on the assumption that some proportion of the genes influencing these traits is fixed, and so the remaining polymorphic risk genes may then be more readily identified within this quieter genetic background, typically using within-breed mapping approaches.

Different genes may be fixed in each breed. Thus, association signals identified in one breed may not be associated in all breeds, unless there is similar intense selection pressure on those genes across breeds. This is evident in the canine ocular disease Progressive Retinal Atrophy. It has a similar presentation in different breeds but can be caused by a variety of mutations. In Irish setters, the condition is due to a single base mutation in *pde6b,* while in collies, it is a 22 base insertion in *rd3* that leads to the same early-onset signs [61]. It is conceivable that the same breed variation will be true for genes involved in behaviour.

Conversely, mutations in the same gene may produce diverse phenotypes in different breeds. Takeuchi et al. [62] examined the canine genome for relationships to behavioural phenotypes. A factor analysis based on descriptors of 81 Labrador retrievers being trained as guide dogs found that polymorphisms in two genes – *comt* and the glutamate receptor (*slc1a2*) – significantly related to a principal component described as ‘activity’. Dogs with the genotype *slc1a2* TT were significantly more active than CT or CC. The same authors examined this *slc1a2* polymorphism in shiba inu and found dogs with the genotype CC were significantly less aggressive to strangers than those with a genotype TC. Those with genotype TT were excluded from the analysis due to the small sample size [63].

Breed and familial predilections for compulsive disorders suggest a genetic basis. For example, bull terriers are prone to spinning or tail chasing [64], while flank sucking is almost exclusively seen in Dobermanns [65]. Both are classified as compulsive disorders but do they involve the same process? The candidate identified by Dodman et al. [66] for flank and blanket sucking in Dobermanns was not found to be associated with tail chasing in bull terriers, Staffordshire bull terriers or German shepherds [67].

It must be acknowledged that several breeds have changed considerably over time. As the selection emphasis for physical or behavioural traits changes, breed phenotypes may shift in response. Svartberg [60] proposes that breed-typical behaviours reflect current selective practices rather than the historical uses of breeds. This suggests that it is possible to breed animals with temperaments that are quite altered from the original breed stock. While this may be desirable for selecting against behavioural disorders, it also promotes consternation among those in the dog-breeding community who value the traditional breed-specific behaviours. Some of the most commonly observed breed ‘splits’ exist between lines of dogs bred for exhibition and those bred for work [68]. Fortunately, population genetics theory suggests that breed-characteristic behaviours should not be ‘lost’ from lines of dogs selected for other traits (such as conformation), unless there is active natural selection against them or unfavourable correlated genetic response from selection on other phenotypes. However, founder effects and drift may lead to fixation for unfavourable alleles at relevant loci.

#### Behaviour and morphotypes

Dogs are unique in their morphological range. For example, across approximately 400 breeds, the variation in canine body size is immense. The Chihuahua reaches a maximum height of 20 cm and weight of 2 kg while the Newfoundland stands at a height of 70 cm and weighs 60 kg. Skull dimensions vary enormously too. Cephalic index (CI: skull width divided by skull length × 100) varies from 37.1 in the borzoi to 101.8 in the French bulldog [69]. This diversity provides opportunities to study correlations between morphology and behaviour. For example, short-skulled dogs are more attentive to pointing signals from humans [70] and more likely to self-groom but less likely to chase [71]. Martìnez et al. [72] found that aggression directed towards people significantly increased as dog size decreased. Similarly, breed height showed strongly significant inverse relationships with behaviours such as mounting persons or objects, touch sensitivity, dog-directed fear, separation-related problems, non-social fear, owner-directed aggression, begging for food, and attachment/attention-seeking [71]. Ley et al. [73] found that dog height and weight were negatively associated with the personality dimension of neuroticism (how nervously they behaved) and positively associated with amicability (how well they tolerated others). These findings are supported by recent association analysis. Correlations were found between loci for physical traits and the behavioural traits identified using C-BARQ in 2,000 dogs [74]. For example, loci for small body size correlated with anxiety/fear traits. The stage is set for further exploration of the genetic determinants of at least some of these associations. Of course, the human side of the equation must also be considered: owner expectations, management and training methods may vary with the size of the dog [75].

#### Heritabilities and breeding programmes

A need to improve efficiency and skill within service-dog programmes has motivated the desire to understand the genetic contribution to canine behaviour in working dogs. Working dogs belong to a diverse group of service areas including assistance (e.g. guide dogs), hunting, herding, livestock protection, defence and detection. The significant cost of training dogs for specialised occupations means that indicators of working success are highly desirable to prevent unnecessary expenditure on training dogs with below-average aptitude for the required tasks. There may also be welfare implications for dogs unsuited to a particular style of training or work.

The emphasis of breeding programmes is on improving the success rates of dogs enrolled in their training programmes [76–79]. A quantitative approach is commonly employed, calculating the heritability of valuable behavioural traits [28, 31, 76, 80–83]. A good example is fear, which has been shown to have a significant impact on training failure among potential guide dogs. As fear and overall training success have similarly robust heritability estimates (0.46 and 0.44 respectively), quantitative genetic methods have been effectively used to improve success rates in guide dog breeding programmes [76, 83].

Most canine breeding programmes are selecting dogs from a single breed to perform a particular function. One of the few exceptions to this is the Swedish Dog Training Centre which breeds and trains both German shepherds (used for guarding or police work) and Labrador retrievers (used as guide dogs). The dogs are raised in the same environment and undergo the same assessments for courage, sharpness, defence drive, prey drive, nerve stability, hardness, temperament, cooperation, affability and gun shyness [82, 84]. The heritability estimates calculated were very similar between the breeds for the first eight of these traits but did differ significantly in the latter two [84]. The heritability estimate of affability (defined as willingness of dog to approach humans) was 0.38 in German shepherds and 0.03 in Labrador retrievers. Gun shyness heritability was calculated at 0.22 in the German shepherds and 0.56 in the Labrador retrievers.

Hunting is another working context for which dogs have been intensively selected to show behaviours such as pointing, searching, pursuit and retrieval. The majority of desired hunting traits have positive genetic correlations [85]. This is useful in a directional selection programme because improvements may be achieved by indirect selection, for example, by using alternative tests that are readily available rather than direct selection for traits that may occur later in life or that may be difficult or expensive to measure. Studies have reported heritability estimates ranging from as low as 0.05 for the ‘search’ trait in Finnish hound [31] up to 0.74 for ‘waiting passively in a group’ in Swedish flat-coated retrievers [85].

Breeding programmes have also assisted the search for causative genes in medical research. The identification of the causative allele for canine narcolepsy was made possible by the establishment of a colony of narcoleptic Dobermanns and Labradors. Twenty years after the colony was established at Stanford University, linkage analysis identified the causative allele on the hypocretin receptor 2 (*hcrtr2*) gene [86]. Hypocretin had not previously been considered a candidate gene.

A classic behavioural experiment spanning three decades studied the genetics of nervousness in English pointers [87, 88]. Two selection lines of dogs were established: one line exhibited extreme responses to noise, avoidance of humans, trembling and catatonia, while the other was a control line of stable temperament. All dogs were exposed to the same environment and learning experiences. Offspring produced from crosses between the two lines were similar to the nervous line, leading Murphree [89] to suggest that the nervous behaviours were inherited in an autosomal dominant manner. The nervous line of pointers had lower body weights, lower weight/height ratios and lower serum IGF-1 levels compared to the normal line [90] and were more susceptible to mange [89]. The group also found that 75% of the nervous dogs suffered from bilateral deafness, although their hearing status did not affect their response to humans [91].

It is worth remembering that heritability calculations are only accurate for that population, in that environment and at that time. Despite this, heritability estimates are still a very useful guide for breeding programmes: the higher the heritability estimate, the more gain will be made by selection. Even traits with modest heritability can demonstrate considerable genetic improvement through selection based on estimated breeding values [27]. The challenge in many breeding programmes is to have sufficient numbers of tested breeding candidates to enable selection intensities that might generate improvement [92].

#### Candidate gene studies

Molecular approaches to the amelioration of behavioural disorders have concentrated on those genes involved in the regulation of common neurotransmitters. Serotonergic receptor genes have been considered candidate genes in many studies looking at panic disorders in humans, but results have been inconsistent. Anxious dogs had significantly higher plasma concentrations of dopamine and serotonin compared with controls [93]. The involvement of serotonin 2A receptors in different canine behavioural disorders has been recently evaluated [94, 95]. Anxious dogs in these studies were shown to have a decreased binding index of 5-HT2A in their right frontal cortex while there was an increased binding index in dogs showing impulsive aggression. This may explain the lower serotonergic metabolite concentrations found in the cerebral spinal fluid of aggressive dogs relative to non-aggressive dogs [96]. A recent study reported lower serotonin concentrations in aggressive English cocker spaniels compared with aggressive dogs of other breeds (318.6 ng/ml compared to 852.77 ng/ml, respectively) [97]. Genes involved in the regulation of serotonin remain the most commonly explored candidate genes in behaviour studies.

Examining similar candidate genes for human-directed aggression in English cocker spaniels identified risk and protective haplotypes in the dopamine receptor D1 (*drd1*), serotonin receptors 1D and 2C (*htr1d* and *htr2c*) and neurotransmitter transporter *slc6a1* [98, 99]. The odds ratio of dogs with a risk haplotype being aggressive to humans compared with those having a protective haplotype varied from 4.4 to 9.0. However, no haplotype demonstrated complete association with the aggression phenotype and research in this population is continuing.

Male dogs are over-represented in cases of canine aggression disorders [21]. A candidate gene study of the androgen receptor gene enabled the detection of three alleles in the trinucleotide (CAG) repeat region in exon 1 in the Japanese Akita inu breed [100]. Male dogs with the shortest allele demonstrated a higher score for owner-directed aggression than male dogs with the longer allele. No association with the allelic length was found in female dogs.

The D4 dopamine receptor gene (*drd4*) has been previously associated with novelty seeking behaviour in humans [101]. Lee et al. [102] examined the association of *drd4* with fearfulness and fearlessness (phenotyped by testing for avoidance of a stranger) in 264 Korean native dogs. Although the results suggested that fear could not be perfectly described by this gene, markers at the D4 receptor were found to significantly predict canine fearfulness. Differing variable number tandem repeats in *drd4* have also been associated with activity and impulsivity in German shepherds [103, 104] and Siberian huskies [105].

#### Genome-wide Association analysis

Molecular genome-wide association analyses have identified single nucleotide polymorphisms that segregate with the boldness-shyness axis in dogs [10, 11]. Chase et al. [106] nominated *drd1* and *igf1* as positional candidate genes for boldness. This is particularly interesting when we recall that the nervous strains of pointers showed lower serum IGF-1 levels. Jones et al. [10] also identified loci with a significant association with herding and pointing.

Researchers studying the genetics underlying canine noise phobia examined the genomes of border collies, Australian shepherds, bearded collies, Belgian shepherds, Belgian Tervurens, Great Danes and German shepherds. Regions on chromosomes 5, 8 and 10 demonstrated moderate association with noise phobia, although none reached genome-wide significance [107]. The genotyping arrays employed in the analysis may have not had sufficient density to detect all association signals in the data. Meanwhile, a comparison of aggressive and non-aggressive golden retrievers using mutation screens, linkage analysis, an association study and a quantitative genetic analysis failed to find evidence linking human-directed aggression with the serotonergic genes *htr1a*, *htr1b*, *htr2a* and *slc6a4* [108]. However, several loci identified during the genome-wide association analysis remain the subjects of ongoing research.

To date, only one study has achieved genome-wide statistical significance identifying a gene relating to a behavioural disorder [66]. In their work on compulsive blanket and flank sucking in Dobermanns, the authors identified a single locus with genome-wide significance within the gene Cadherin 2 (*cdh2*), a widely expressed gene involved in pre- and post-synaptic adhesion. The risk genotypes (TT or CT) were more frequent in the severe phenotypes. However, polygenic inheritance is still suspected. This modest amount of success speaks to the underlying genetic complexity of such disorders in all species.

## Conclusions

The influence of genetics on both desirable and undesirable behavioural phenotypes is considerable and, consequently, many traits relating to behaviour should be amenable to selection. Significant improvement in the efficiency of selection for desirable behavioural traits is possible when objective standardised phenotyping is used. Additional efficiency can be gained by employing modern animal-breeding technologies, such as best-linear unbiased predictor-based estimated breeding values. In this review we demonstrate that programmes that have undertaken rigorous standardised phenotyping of working dogs and that have also employed proven quantitative genetic approaches have already demonstrated impressive genetic progress.

Medical genetic research in all species is progressing rapidly. While interest and endeavour in researching behavioural genetics is certainly present, and some exciting advances have been made, progress has been slow. By using standardised phenotyping, standardised terminology and encouraging collaboration among research groups, many of the current limitations to behavioural genetics research will be overcome, allowing us to improve the lives of our closest companions and better understand human psychiatric disorders.
